# Functional role of opponent, dopamine modulated D1/D2 plasticity in reinforcement learning

**DOI:** 10.1186/1471-2202-14-S1-P199

**Published:** 2013-07-08

**Authors:** Jenia Jitsev, Nobi Abraham, Abigail Morrison, Marc Tittgemeyer

**Affiliations:** 1 Cortical Networks Group, Max-Planck-Institute for Neurological Research, Gleueler Str. 50, 50931 Cologne, Germany; 2 Functional Neural Circuits Group, Institute of Neuroscience and Medicine (INM-6), Computational and Systems Neuroscience, Research Center Jülich, 52425 Jülich, Germany; 3 Simulation Lab Neuroscience, Bernstein Facility Simulation and Database Technology (BFSD), Research Center Jülich, 52425 Jülich, Germany

## 

The basal ganglia network is thought to be involved in adaptation of organism's behavior when facing its positive and negative consequences, that is, in reinforcement learning. It has been hypothesized that dopamine (DA) modulated plasticity of synapses projecting from different cortical areas to the input nuclei of the basal ganglia, the striatum, plays a central role in this form of learning, being responsible for updating future outcome expectations and action preferences. In this scheme, DA transmission is considered to convey a prediction error signal that is generated if internal expectations do not match the outcomes observed after action execution. So far, there has been no satisfying model for what neural circuits computing this signal within the basal ganglia may look like, how this computation is performed and what is the mechanistic role of DA release in adapting the system towards optimal behavior in a given task.

Aiming towards a model of a canonical circuit for learning task-conform behavior from both reward and punishment, we extended a previously introduced spiking actor-critic network model of the basal ganglia [1] to contain the segregation of both the dorsal (actor) and ventral (critic) striatum into populations of D1 and D2 medium spiny neurons (MSNs). This segregation allows explicit, separate representation of both positive and negative expected outcomes by the distinct populations in the ventral striatum. The positive and negative components of expected outcome were fed to dopamine (DA) neurons in SNc/VTA region, which compute and signal reward prediction error by DA release. Based on recent experimental work [2], DA level was assumed to modulate plasticity of D1 and D2 synapses in opposing way, inducing LTP on D1 and LTD on D2 synapses if being high and vice versa if being low. Crucially, this form of opponent plasticity implements temporal-difference (TD)-like update of both positive and negative outcome expectations separately and performs appropriate action selection adaptation.

We implemented the network in the NEST simulator [3] using leaky integrate-and-fire spiking neurons and designed a battery of experiments involving application of reward and punishment in various grid world tasks. In each task, an agent had to explore the states and learn to maximize the total reward obtained. Number of states, magnitudes and delays of reward and punishment were manipulated across different tasks. We demonstrate that across the tasks the network can learn both to approach the delayed rewards while consequently avoiding punishments, the latter posing severe difficulties for the previous model without D1/D2 segregation [1]. Thus, the spiking neural network model highlights the functional role of D1/D2 MSN segregation within the striatum in implementing appropriate TD-like learning from both reward and punishment and explains necessity for opponent direction of DA-dependent plasticity found at synapses converging on distinct striatal MSN types. This modeling approach can be extended in the future work to study how abnormal D1/D2 plasticity may lead to a reorganization of the basal ganglia network towards pathological, dysfunctional states, like for instance those observed in Parkinson disease under condition of progressive dopamine depletion.
